# Dr. Herman B. Singer

**Published:** 1912-11

**Authors:** 


Dr. Herman B. Singer of Buffalo, died Oct. 26, 1912. He was born in 1864, graduated at Leipzig in 1893 and has been a resident of Buffalo for many years, being a member of the Academy of Medicine, the County Society and other organizations.

